# Heart rate response during 6-minute walking testing predicts outcome in operable chronic thromboembolic pulmonary hypertension

**DOI:** 10.1186/s12890-016-0260-y

**Published:** 2016-07-04

**Authors:** Manuel Jonas Richter, Katrin Milger, Khodr Tello, Philipp Stille, Werner Seeger, Eckhard Mayer, Hossein A. Ghofrani, Henning Gall

**Affiliations:** Department of Pneumology, Kerckhoff Heart, Rheuma and Thoracic Center, Bad Nauheim, Germany; Department of Internal Medicine, Justus-Liebig-University Giessen, Universities of Giessen and Marburg Lung Center (UGMLC), Member of the German Center for Lung Research (DZL), Giessen, Germany; Department of Internal Medicine V, University of Munich, Comprehensive Pneumology Center, member of the German Center for Lung Research (DZL), Munich, Germany; Department of Thoracic Surgery, Kerckhoff Heart, Rheuma and Thoracic Center, Bad Nauheim, Germany; Department of Medicine, Imperial College London, London, UK

**Keywords:** Oxygen desaturation, Heart rate response, Chronic thromboembolic pulmonary hypertension, Pulmonary endarterectomy

## Abstract

**Background:**

Six-minute walk test (6MWT) is routinely performed in chronic thromboembolic pulmonary hypertension (CTEPH) before pulmonary endarterectomy (PEA). However, the clinical relevance of heart rate response (ΔHR) and exercise-induced oxygen desaturation (EID) during 6MWT is remaining unknown.

**Methods:**

Patients undergoing PEA in our center between 03/2013-04/2014 were assessed prospectively with hemodynamic and exercise parameters prior to and 1 year post-PEA. Patients with symptomatic chronic thromboembolic disease (mean pulmonary artery pressure (mPAP) <25 mmHg) and clinical relevant obstructive pulmonary disease were excluded. The following definitions were used: ΔHR = (peak HR - resting HR), percent heart rate reserve (HRR) = (peak HR –rest HR)/(220 - age - rest HR) x 100 and EID = SpO_2_ ≤88 %.

**Results:**

Thirty-seven patients (of 116 patients screened) with mPAP of 43.2 ± 8.7 mmHg, pulmonary vascular resistance (PVR) of 605.5 ± 228.7 dyn*s/cm^5^, cardiac index (CI) of 2.4 ± 0.5 l/min/m^2^ and a 6MWT-distance of 404.7 ± 148.4 m and a peak VO_2_ of 12.3 ± 3.4 ml/min/kg prior to PEA were included. Baseline ΔHR during 6MWT was significantly associated with PVR 1 year post-PEA using linear regression analysis (*r* = 0.43, *p* = 0.01). Multivariate analysis indicated an association of HRR during 6MWT and residual PH with a hazard ratio of 1.06 (95 % Confidence interval for hazard ratio 0.99–1.14, *p* = 0.08). EID was observed commonly during 6MWT but no correlations to outcome parameters were found.

**Conclusions:**

This is the first prospective study to describe an association of ΔHR during 6MWT with pulmonary hemodynamics 1 year post-PEA. Our preliminary results indicate that HRR derived from 6MWT is of clinical significance. EID was commonly observed, albeit failed as a significant prognostic factor.

## Background

Chronic thromboembolic pulmonary hypertension (CTEPH) is defined by an elevation of mean pulmonary arterial pressure (mPAP) and pulmonary vascular resistance (PVR) caused by unresolved pulmonary vascular obstruction due to recurrent embolism [1, 2]. Mechanical obstruction in proximal parts of the pulmonary vascular system and secondary small-vessel arteriopathy in the non-obstructed areas are causes of disease progression and lead to extensive right ventricle (RV) dysfunction, loading and failure [1, 2]. Pulmonary endarterectomy (PEA) is the gold standard in case of surgical accessible CTEPH and offers a potential curative treatment with an improved functional outcome and high survival rates [3, 4]. Predictors of favorable outcome after PEA are important in daily clinical practice and include pre-operative forced expiratory volume in 1 s (FEV1), heart-type fatty acid-binding protein (H-FABP) and cardiac index (CI) [5, 6]. In addition, 6-minute walk testing (6MWT) with measurement of the distance covered (6MWD) is performed routinely before and after PEA as a tool to assess disease severity, functional capacity or long-term outcome [7, 8]. Moreover, preoperative 6MWD correlates with 3-month survival after PEA [9]. However, the prognostic relevance of additional parameters derived from the 6MWT were not evaluated in detail before. Presently, it is unknown whether exercise-induced oxygen desaturation (EID) or heart rate response (ΔHR) during 6MWD associate with disease severity, functional or hemodynamic outcome in operable CTEPH. Nevertheless, patients with CTEPH frequently display significant EID with inadequate increase in heart rate (HR) during exercise [10]. Previously, EID has been related to reduced exercise performance and increased mortality in patients with chronic obstructive pulmonary disease (COPD) and pulmonary fibrosis [11, 12], whereas ΔHR in pulmonary arterial hypertension (PAH) was analyzed in the context of baseline exercise capacity and functional improvements under targeted therapy [13]. Furthermore, chronotropic incompetence derived from heart rate reserve (HRR) was identified as an important and independent predictor of mortality in population based studies [14]. Moreover, parameters such as peak oxygen uptake (VO_2_), peak systolic and diastolic blood pressure derived from cardio pulmonary exercise testing (CPET) are better correlated with functional class and prognosis than resting hemodynamic parameters in PAH [15]. So far, the significance of ΔHR and EID during 6MWD in comparison to CPET is remaining unknown in CTEPH.

We hypothesized that EID during 6MWD in CTEPH might be of prognostic relevance and related to the extent of dyspnea. Moreover, we speculated that the ΔHR derived from the 6MWT reflects the inappropriate response of the RV with insufficient increase of the cardiac output (CO) to exercise, as it was shown for CPET [16]. Therefore, our aim was to prospectively investigate ΔHR and EID during 6MWT in patients with operable CTEPH and their associations with clinical symptoms and hemodynamic outcome 1 year post-PEA. In addition, we aim to compare ΔHR and EID assessed during 6MWT with CPET prior PEA in regard of their predictive significance.

## Methods

### Patients

**Fig. 1:**
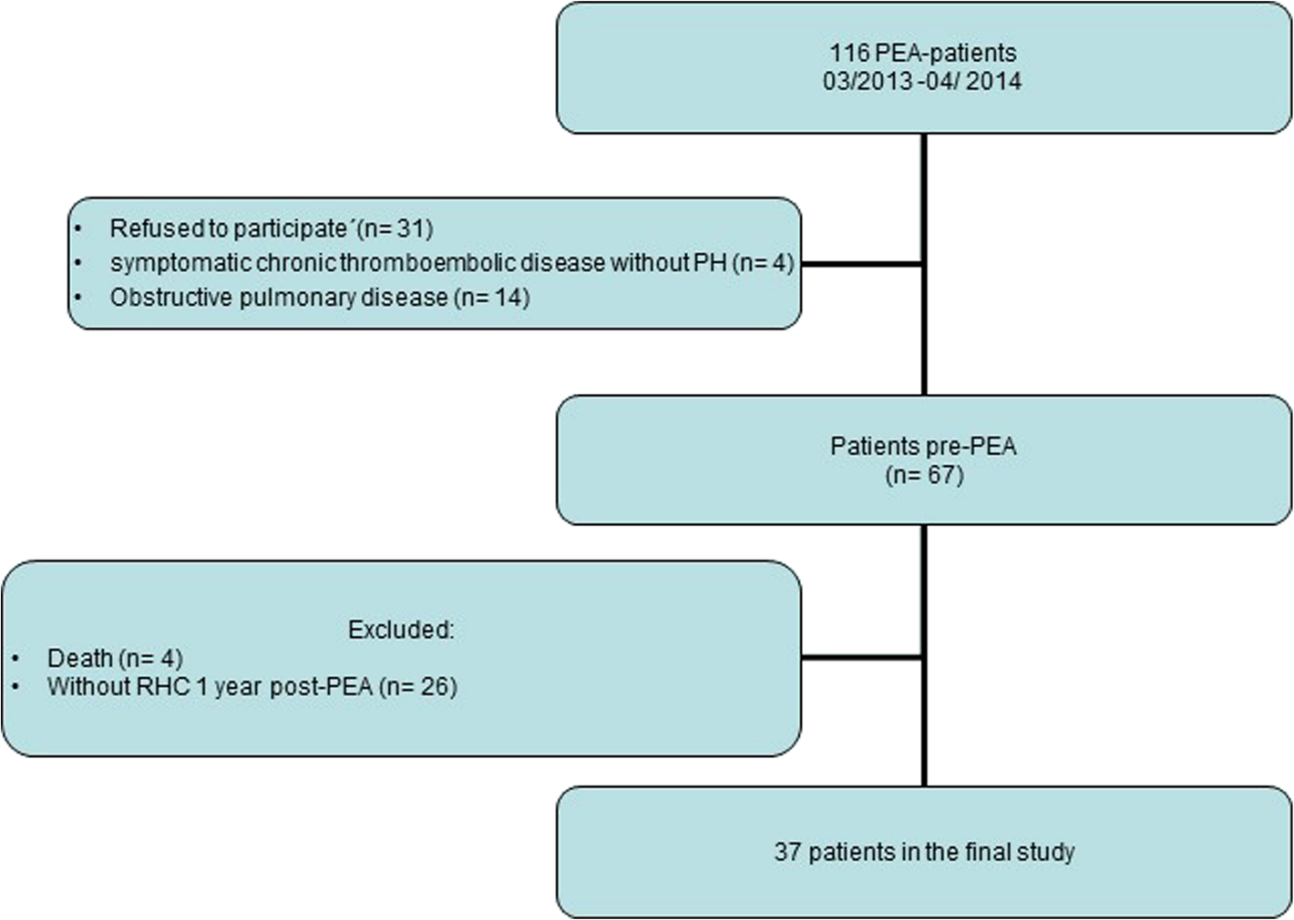
Flow chart of patient selection. RHC: right heart catheterization, PEA: pulmonary endarterectomy, PH: pulmonary hypertension

All CTEPH patients undergoing PEA between March 2013 and April 2014 at the Department of Thoracic surgery, Kerckhoff-Clinic, Bad Nauheim, Germany were prospectively screened. After exclusion, 67 patients entered the study pre-PEA, while only patients with complete baseline and 1 year post-PEA hemodynamic data were analyzed (*n* = 37) (Fig. 1). Baseline and follow-up right heart catheter (RHC) were not mandatorily performed in-house, as the Kerckhoff-Clinic is a national referral center. Patients with symptomatic chronic thromboembolic disease (mean pulmonary artery pressure <25 mmHg at baseline [17]), obstructive pulmonary disease (forced expiratory volume in 1 s/vital capacity (FEV1/VC) ≤70 %) were excluded.

CTEPH was diagnosed according to current guidelines [18] and operability was assessed by a multidisciplinary board including pulmonary physicians, PEA surgeons and pulmonary radiologists. All patients received oral anticoagulants for at least 3 months and underwent PEA according to the protocol of the Kerckhoff-Clinic [19]. Residual PH 1 year post-PEA were defined by mPAP >25 mmHg and PVR >240 dyn*s/cm^5^ at rest [20] while CTEPH type was classified by the surgical specimen as described previously [21].

All included patients gave written, informed consent, and the study was approved by the by the ethics committee of the Faculty of Medicine at the University of Giessen (Approval No. 67/14).

The following definitions were used: ΔHR = (peak HR - resting HR) [22], HRR = (peak HR –rest HR)/(220 - age - rest HR) x 100 [23] and EID = oxygen pulse saturation (SpO_2_) ≤88 % [12, 24].

### 6-minute walk test

All patients performed 6MWT at the Kerckhoff-Clinic according to current guidelines [25]. Patients were instructed to walk at their own pace while standard phrases were communicated [25]. HR and oxygen pulse saturation (SpO_2_) using pulse oximetry (Oximax™, N-65™, Covidien AG, USA) were measured at baseline and every minute until minute 6. In addition, patients were asked to quantify their sensation of dyspnea with the BORG scale [26]. As described previously, the respiratory therapist checked that the pulse oximeter had an acceptable signal before beginning all tests and carefully instructed the patient [12]. Patients already receiving supplemental oxygen at rest performed 6MWT with their current oxygen dosage. 6MWD was defined as the maximal achieved walk distance in m at room air or oxygen-supplemented.

### Cardio-pulmonary exercise testing

Prior PEA patients performed a symptom-limited incremental CPET using a ramp protocol with an incremental rate of 5 to 15 W/min judged by the operator (Masterscreen CPX®, Carefusion®), according to current recommendations [27]. Patients receiving supplemental oxygen and exhibited a resting SpO_2_ less than 88 % were excluded. Patients were asked to exercise up to their individual limit, while exercise was terminated due to exercise-limiting symptoms by the patient. Dead space ventilation (V_D_/V_T_) was calculated using the Bohr- formula, while absolute dead space (V_D_) was assessed in milliliters [28].

### Baseline parameters

Hemodynamic, laboratory, echocardiography data were collected before and after PEA in all included patients. Right heart echocardiography assessed pulmonary artery systolic pressure (PASP) and tricuspid annular plane systolic excursion (TAPSE) according to current recommendations [29].

### Statistical analysis

Data are presented as mean ± standard deviation (SD) for normally distributed parameters or median [interquartile range (IQR)]. As appropriate the 2-tailed *T*-test, paired *T*-test, Wilcoxon Signed Rank test, Mann-Whitney-*U*-test or Pearson Chi-Square test was used to test for differences between groups. Linear regression analysis was performed between follow-up hemodynamic parameters and baseline 6MWT and CPET characteristics. In addition, all baseline parameters were analyzed univariate in a logistic regression model with residual PH as dependent variable. Then all parameters of the univariate analysis with a *p*-value < 0.15, were entered into a backward stepwise multivariate logistic regression model to predict residual PH 1 year post-PEA. *P*-value of < 0.10 was considered statistically significant in the multivariate analyses. Statistical analysis was performed using SPSS, version 21.0 (IBM, Armonk, NY).

## Results

### Baseline

Thirty-seven CTEPH patients with a mean age of 61 ± 12 years and a body mass index of 27.9 ± 5.8 kg/m^2^ mostly in WHO functional class III were included. Patients showed impaired pulmonary hemodynamics with a precapillary pattern, substantially reduced CI and elevated PVR before PEA. Concomitant right heart echocardiography showed an elevated PASP and a reduced TAPSE. Lung function testing revealed no significant obstructive or restrictive ventilatory abnormalities. During 6MWT peak SpO_2_ decreased to 88.8 ± 5.6 % with a Δ SpO_2_ of - 5.1 ± 4.4 %, HR increased to a mean peak of 117.1 ± 18.8 beats*min^−1^ while ΔHR was 32.6 ± 14.7 beats*min^−1^ and HRR was 45.1 ± 20.6 %. Sensation of dyspnea as assessed by the BORG score showed a substantial increase (Δ BORG 4.4 ± 2.2). In total, 9 patients underwent the 6MWT with supplemental oxygen (Table 1). In total, 34 patients performed CPET displaying an impaired exercise capacity while V_D_ and V_D_/V_T_ exhibited a significant increase during exercise. During CPET peak SpO_2_ decreased to 89.3 ± 1.4 % with a ΔSpO_2_ of - 4.9 ± 5.8 %, HR increased to a mean peak of 120.7 ± 21.3 beats*min^−1^ while ΔHR was 47.9 ± 19.4 beats*min^−1^ and HRR was 54.7 ± 22.1 %.

**Table 1:** Baseline characteristics

	Baseline	One year post-PEA	*p*-value
Patients, *n*	37		
Male/female	20/17		
Age (y)	61 ± 12		
BMI (kg/m^2^)	27.9 ± 5.8		
WHO functional class, (%)		b	0.7
I	none	29.6	
II	27	48.1	
III	62.2	22.2	
IV	10.8	None	
6MWT			
6MWD (m)	404.7 ± 148.4	453.4 ± 126.8	0.1
Rest SpO_2_ (%)	93.9 ± 2.7	95.6 ± 2.9	0.043
Peak SpO2 (%)	88.8 ± 5.6	92.2 ± 4.5	0.003
Δ SpO2 (%)	−5.1 ± 4.4	−3.5 ± 4.1	0.15
Rest HR (beats^b^min-1)	83.8 ± 14.4	83.5 ± 13.9	0.98
Peak HR (beats^b^min-1)	117.1 ± 18.8	107.8 ± 17.1	0.041
Δ HR (beats^b^min-1)	32.6 ± 14.7	24.3 ± 12.9	0.038
HRR (%)	45.1 ± 20.6	33.4 ± 16.7	0.022
Δ Borg	4.4 ± 2.2	3.1 ± 2.4	0.006
Supplemental Oxygen (*n*, %)	9 (24.3)	3 (8.1)^b^	0.33
Lung function			
FEV1 (% pred.)	88.7 ± 13.8	88.2 ± 12.5	0.76
FEV1/FVC (% pred.)	96 ± 10.3	90.6 ± 12.1	0.05
TLC (% pred.)	99 ± 13.5	104.8 ± 16.8	0.11
VC (% pred.)	91.6 ± 13.7	94 ± 12.7	0.34
RHC			
mPAP (mm Hg)	43.2 ± 8.7	28.9 ± 10.1	0.001
RAP (mm Hg)	5.9 ± 4.1	7.2 ± 4.3	0.15
PVR (dyne^b^s/cm^5^)	605.5 ± 228.7	328.1 ± 241.4	0.001
CI (l/min/m^2^)	2.4 ± 0.5	2.7 ± 1.3	0.048
PAWP (mm Hg)	9.5 ± 4.6	10.6 ± 4.5	0.19
Echocardiography			
TAPSE (mm)	17.2 ± 4.3	17.5 ± 3.2	0.62
PASP (mm Hg)	69.8 ± 25.1	56.8 ± 23.7	0.041
CPET^c^			
Peak VO_2_ (ml/min/kg)	12.3 ± 3.4	14.2 ± 4.2	0.33
Rest V_D_/V_T_	35.2 ± 7.6	32 ± 5.8	0.77
Peak V_D_/V_T_	39.5 ± 8.7	35 ± 11.9	0.6
Rest V_D_, L	0.35 ± 0.17	0.29 ± 0.11	0.39
Peak V_D_, L	0.65 ± 0.25	0.72 ± 0.21	0.55
Rest SpO_2_ (%)	94.2 ± 1.7	95.1 ± 1.4	0.10
Peak SpO2 (%)	89.3 ± 1.4	90.7 ± 4.8	0.86
Δ SpO2 (%)	−4.9 ± 5.8	−4.4 ± 4.3	0.69
Rest HR (beats^b^min-1)	72.8 ± 12.2	81.2 ± 11.2	0.15
Peak HR (beats^b^min-1)	120.7 ± 21.3	122.9 ± 19.6	0.25
Δ HR (beats^b^min-1)	47.9 ± 19.4	41.7 ± 16.3	0.72
HRR (%)	54.7 ± 21.1	53.7 ± 20.2	0.92
Co-morbidities, *n* (%)			
Hypertension	24 (64.9)	Unchanged	
Coronary heart disease	4 (10.8)	Unchanged	
Renal insuffiency	4 (10.8)	Unchanged	
Jamieson-Type, %			
I	31		
II	31		
III	38		
Laboratory			
NT-proBNP (pg/ml)	488.2 [1004]	245.0 [422]^a^	0.006

Values represent mean ± SD or median [IQR]. pred.: predicted, ^a^ = Wilcoxon Signed Rank test, ^b^ = Pearson Chi-Square test, ^c^ *n* = 34. *CI* cardiac index, *mPAP* mean pulmonary arterial pressure, *PVR* pulmonary vascular resistance, *RAP* right atrial pressure, *PAWP* pulmonary arterial wedge pressure, *TAPSE* tricuspid annular plane systolic excursion, *PASP* pulmonary artery systolic pressure, *6MWD* six-minute walking distance, *VO*_*2*_ oxygen uptake, *WHO* World Health Organization, *NT-proBNP* N-terminal fragment of pro-brain natriuretic peptide, *V*_*D*_ absolute dead space, *V*_*D*_ */V*_*T*_ dead space ventilation, *HR* heart rate, *SpO*_*2*_ oxygen pulse saturation, *RHC* right heart catheter, *CPET* cardio-pulmonary exercise testing, *HRR* heart rate reserve, *VC* vital capacity, *FRC* functional residual capacity, *TLC* total lung capacity, *FEV1* forced expiratory volume in 1 s

### One year post-PEA

Hemodynamic and functional parameters significantly improved 1 year post-PEA as compared to baseline. Patients presented mostly in WHO functional class I and II, resting and peak SpO_2_ were significantly higher during 6MWT and peak HR, ΔHR and HRR and NT-proBNP were significantly lower 1 year post-PEA. A trend towards decrease was observed in resting V_D_/V_T_ and V_D_ during CPET (Table 1). In total, 21 patients presented with residual PH displaying significantly elevated mPAP, PVR, NT-proBNP and reduced CI in comparison with non-residual PH. In addition, patients with residual PH presented to a higher degree in WHO functional class III and showed, in comparison with non-residual PH, a significant lower rest and peak SpO_2_ during 6MWT. HRR was significantly higher in patients with residual PH while ΔHR showed a trend to higher values. Dyspnea as measured by BORG score was significantly increased in patients with residual PH, while the administration of supplemental oxygen did not differ between groups (Table 2). In comparison with non-residual PH no significant differences of peak VO_2_, SpO_2_, HR or HHR derived from CPET were shown.

**Table 2:** Parameters 1 year post-PEA according to non-residual and residual PH

	Non-residual PH	Residual PH	*p*-value
Patients, *n* (%)	16 (43.2)	21 (56.8)	
WHO functional class, (%)		a	0.002
I	46.2	None	
II	53.8	57.1	
III	None	42.9	
IV	None	None	
6MWT			
6MWD (m)	487.6 ± 72.1	432.4 ± 148.9	0.26
Rest SpO_2_ (%)	97 ± 1.9	94.8 ± 3.1	0.05
Peak SpO2 (%)	94.3 ± 4.4	90.8 ± 4.1	0.05
Δ SpO2 (%)	−2.7 ± 3.8	−3.9 ± 4.4	0.47
Rest HR (beats^a^min-1)	83.9 ± 15.1	83.2 ± 13.9	0.9
Peak HR (beats^a^min-1)	101.6 ± 15.2	111.5 ± 17.6	0.2
Δ HR (beats^a^min-1)	17.8 ± 6.3	28.3 ± 14.4	0.06
HRR (%)	24.3 ± 11.5	28.3 ± 14.4	0.05
Δ Borg	1.7 ± 1.9	3.9 ± 2.4	0.02
Supplemental Oxygen (*n*, %)	1 (6.3)	2 (9.5)	-
Lung function			
FEV1 (% pred.)	93.9 ± 12.8	83.9 ± 10.6	0.02
FEV1/FVC (% pred.)	95.8 ± 13.9	86.7 ± 9.2	0.03
TLC (% pred.)	104.2 ± 15.2	105.2 ± 18.3	0.87
VC (% pred.)	96.8 ± 14.0	92.1 ± 11.6	0.3
RHC			
mPAP (mm Hg)	19.7 ± 3.2	36 ± 7.5	0.001
RAP (mm Hg)	5.5 ± 2.4	8.7 ± 5.1	0.04
PVR (dyne^a^s/cm^5^)	218.3 ± 280.9	415.9 ± 163.7	0.012
CI (l/min/m^2^)	2.9 ± 0.4	2.6 ± 0.6	0.09
PAWP (mm Hg)	8.9 ± 3.8	12.2 ± 4.7	0.037
Echocardiography			
TAPSE (mm)	18.3 ± 2.7	16.7 ± 3.6	0.2
PASP (mm Hg)	50.6 ± 29.2	59.5 ± 21.9	0.5
CPET^b^			
Peak VO_2_ (ml/min/kg)	15.2 ± 4.5	13.3 ± 3.9	0.36
Rest V_D_/V_T_	30.3 ± 5.1	35.3 ± 6.4	0.35
Peak V_D_/V_T_	27.7 ± 6.1	41.2 ± 12.1	0.13
Rest V_D_, L	0.22 ± 0.07	0.37 ± 0.09	0.09
Peak V_D_, L	0.63 ± 0.1	0.78 ± 0.26	0.39
Rest SpO_2_ (%)	95.1 ± 1.3	93.7 ± 1.8	0.10
Peak SpO2 (%)	89.1 ± 7.3	89.6 ± 4.6	0.86
Δ SpO2 (%)	−5.2 ± 5.4	−3.6 ± 3.3	0.69
Rest HR (beats^a^min-1)	76.1 ± 12.4	69.9 ± 11.7	0.15
Peak HR (beats^a^min-1)	125.3 ± 22.4	116.6 ± 19.9	0.25
Δ HR (beats^a^min-1)	49.2 ± 19.3	46.7 ± 19.9	0.72
HRR (%)	55.1 ± 21.1	54.3 ± 21.8	0.92
Jamieson-Type, (%)		a	0.15
I	14.2	46.7	
II	42.9	20	
III	42.9	33.3	
Laboratory			
NT-proBNP (pg/ml)	175.0 [56–259]	389.0 [95–703]	0.18

Values represent mean ± SD or median [IQR]. ^a^ = Pearson Chi-Square test, ^b^ *n* = 34. For abbreviations see Table 1

### Clinical relevance of exercise-induced oxygen desaturation

**Fig. 2:**
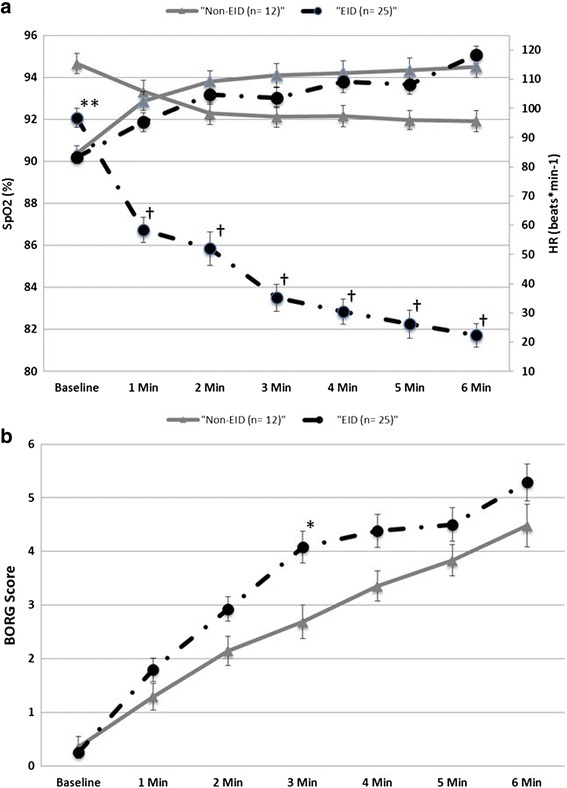
**a** Heart rate (HR) and SpO_2_ during 6MWT according to exercise-induced oxygen desaturation (EID). Data are presented as mean ± standard error of means. **p = 0.002, ^†^ *p* < 0.001 versus non-EID. (*black line* = EID; *grey line* = non-EID; ▲= SpO2; ●= HR). **b** BORG score during 6MWT according to exercise-induced oxygen desaturation (EID). Data are presented as mean ± standard error of means. *p = 0.02 versus non-EID. (*black line* = EID; *grey line* = non-EID)

Baseline characteristics were analyzed according to the presence of EID during 6MWT, while pulmonary hemodynamics, exercise capacity, V_D_/V_T_ and Jamieson-Type showed no significant differences in comparison. Interestingly, 12 out of 37 patients exhibited EID with a ΔSpO_2_ of 10 ± 1.1 % (Table 3), while a rapid continuous desaturation was evident already starting at minute one of the 6MWT (Fig. 2a). BORG score at minute 3 was significantly higher in patients with EID, albeit ΔBORG and peak BORG score showed a trend to higher values in EID (Fig. 2b).

**Table 3:** Parameters at baseline according to exercise-induced oxygen desaturation during 6MWT

	Baseline		
	EID	Non-EID	*p*-value
Patients, *n* (%)	12 (32.4)	25 (67.6)	
WHO functional class, (%)	a		0.09
II	33.3	24	
III	41.7	72	
IV	25	4	
6MWT			
6MWD (m)	448.9 ± 206.8	383.6 ± 109.5	0.22
Rest SpO_2_ (%)	92.0 ± 2.2	94.8 ± 2.1	0.002
Peak SpO_2_ (%)	81.9 ± 4.4	92.1 ± 2.3	0.001
Δ SpO2 (%)	10.0 ± 1.1	2.7 ± 0.4	0.02
Rest HR (beats^a^min-1)	83.5 ± 16.4	84 ± 13.7	0.9
Peak HR (beats^a^min-1)	120.6 ± 20.7	115.5 ± 18.1	0.45
Δ HR (beats^a^min-1)	30.5 ± 5.1	30.4 ± 2.4	0.82
HRR (%)	49.5 ± 8.1	42.5 ± 4	0.53
Δ Borg	4.7 ± 0.7	4.3 ± 0.5	0.52
Supplemental Oxygen (*n*, %)	4 (33.3)	5 (20) ^a^	0.38
Lung function			
FEV1 (% pred.)	83.6 ± 11.9	90.9 ± 14.1	0.15
FEV1/FVC (% pred.)	98.6 ± 10.2	94.8 ± 10.3	0.31
TLC (% pred.)	94.8 ± 16.3	100.8 ± 12.1	0.24
VC (% pred.)	85.8 ± 10.3	94.1 ± 14.4	0.1
RHC			
mPAP (mm Hg)	43.8 ± 8.2	42.9 ± 9.0	0.77
RAP (mm Hg)	4.1 ± 2.7	6.6 ± 4.4	0.8
PVR (dyne^a^s/cm^5^)	640.1 ± 231.0	587.4 ± 230.5	0.53
CI (l/min/m^2^)	2.5 ± 0.6	2.4 ± 0.5	0.7
PAWP (mm Hg)	9.3 ± 2.8	9.7 ± 5.4	0.8
Echocardiography			
TAPSE (mm)	16.6 ± 4.3	17.4 ± 4.3	0.63
PASP (mm Hg)	77.2 ± 30.7	66.2 ± 21.7	0.24
CPET^c^			
Peak VO_2_ (ml/min/kg)	13.1 ± 1.3	12.1 ± 0.6	0.65
Rest V_D_/V_T_	33.0 ± 7.3	36.0 ± 8	0.48
Peak V_D_/V_T_	39.0 ± 12.0	40.0 ± 7.3	0.78
Rest V_D_, L	0.41 ± 0.22	0.34 ± 0.14	0.4
Peak V_D_, L	0.67 ± 0.33	0.65 ± 0.23	0.9
Jamieson-Type, (%)	^a^		0.72
I	25	32	
II	50	26	
III	25	42	
Laboratory			
NT-proBNP (pg/ml)	414.0 [1260.2]	836.7 [1521]^b^	0.71

Values represent mean ± SD or median [IQR]. ^a^ = Pearson Chi-Square test, ^b^ = Mann-Whitney *U* Test, ^c^ *n* = 34.; For abbreviations see Table 1

There were no significant associations between rest SpO_2_, peak SpO_2_ or ΔSpO_2_ with hemodynamic or functional parameters 1 year post-PEA during 6MWT and CPET (data not shown).

Univariate logistic regression analysis revealed that baseline peak SpO_2_ or Δ SpO_2_ during 6MWT were not associated with the presence of residual PH. Univariate analysis related rest SpO_2_ during 6MWT to residual PH with a hazard ratio of 0.8 (95 % Confidence interval for hazard ratio 0.6–1.06, *p* = 0.11). However, additional stepwise backward multivariate analysis showed that rest SpO_2_ derived from 6MWT was not independently associated with the hemodynamic outcome 1 year post-PEA. In addition, rest SpO_2_, peak SpO_2_ or ΔSpO_2_ derived from CPET failed as prognostic markers using univariate analysis (Table 4).

**Table 4:** Baseline parameters as predictors of residual PH 1 year post PEA

	Univariate model		Multivariate model^*^	
	Hazard ratio (95 % confidence interval)	*p*-value	Hazard ratio (95 % confidence interval)	*p*-value
6MWT				
6MWD (m)	1.01 [0.99–1.01]	0.44	-	-
Rest SpO_2_ (%)	1.25 [0.94–1.67]	0.11	-	-
Peak SpO2 (%)	0.96 [0.85–1.08]	0.46	-	-
Δ SpO2 (%)	0.99 [0.86–1.16]	0.96	-	-
Rest HR (beats*min-1)	0.99 [0.94–1.03]	0.6	-	-
Peak HR (beats*min-1)	1.03 [0.99–1.06]	0.19	-	-
Δ HR (beats*min-1)	1.10 [1.03–1.2]	0.009	-	-
HRR (%)	1.06 [1.02–1.1]	0.01	1.06 [0.99–1.14]	0.08
CPET				
Rest SpO_2_ (%)	1.81 [0.86–3.76]	0.12	-	-
Peak SpO2 (%)	1.02 [0.86–1.20]	0.84	-	-
Δ SpO2 (%)	1.05 [0.86–1.27]	0.66	-	-
Rest HR (beats*min-1)	0.96 [0.90–1.02]	0.16	-	-
Peak HR (beats*min-1)	0.98 [0.95–1.01]	0.25	-	-
Δ HR (beats*min-1)	0.99 [0.96–1.03]	0.71	-	-
HRR (%)	1.00 [0.97–1.03]	0.91	-	-
Peak VO_2_ (ml/min/kg)	0.95 [0.78–1.17]	0.63	-	-
RHC				
mPAP (mm Hg)	1.07 [0.98–1.16]	0.13	-	-
RAP (mm Hg)	1.01 [0.83–1.2]	0.96	-	-
PVR (dyne*s/cm^5^)	1.01 [0.99–1.01]	0.19	-	-
CI (l/min/m^2^)	1.10 [0.3–4.01]	0.89	-	-
PAWP (mm Hg)	1.03 [0.89–1.2]	0.67	-	-
Echocardiography				
TAPSE (mm)	0.90 [0.77–1.06]	0.2	-	-
PASP (mm Hg)	1.03 [0.99–1.07]	0.06	-	-
Other				
Nt-pro BNP (pg/ml)	1.00 [0.99–1.01]	0.97	-	-
WHO functional class	-	0.39	-	-
Jamieson-Type				
• I	Reference		Reference	
• II	4.2 [0.6–30.1]	0.15	8.9 [0.7–116.4]	0.096
• III	1.67 [0.28–3.57]	0.58	5.02 [3.23–100]	0.25

For abbreviations see Table 1. ^*^: backward stepwise logistic regression including variables with a *p*-value <0.15 in the univariate model

### Clinical relevance of heart rate response

**Fig. 3:**
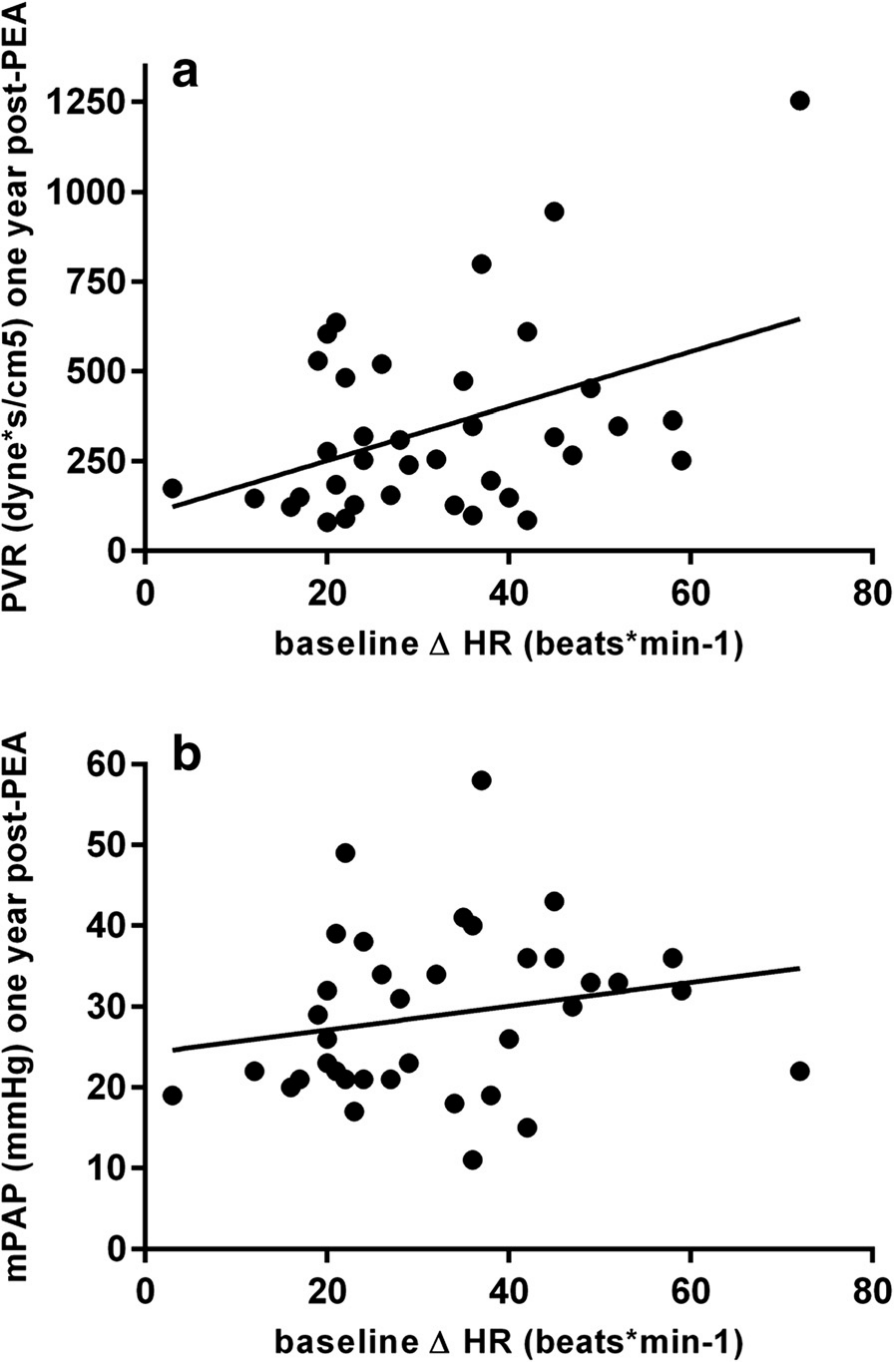
Associations between baseline Δ HR and PVR (*r* = 0.43, *p* = 0.01) (**a**) and mPAP (*r* = 0.21, *p* = 0.23) (**b**) 1 year post-PEA. For abbreviations see Table 1

During 6MWT an initial steep and then flattened HR response was observed (Fig. 2a). Baseline ΔHR during 6MWT was significantly associated with PVR 1 year post-PEA (*r* = 0.43, *p* = 0.01) (Fig. 3a). However, using linear regression analysis ΔHR showed no significant associations with mPAP (Fig. 3b) or other pulmonary hemodynamic parameters 1 year post-PEA (data not shown). There were no significant associations of rest HR, peak HR or HRR with hemodynamic parameters 1 year post-PEA (data not shown). Interestingly, HR parameters derived from CPET were not significantly associated with hemodynamic parameters 1 year post-PEA (data not shown).

Univariate logistic regression analysis revealed that baseline ΔHR and HRR during 6MWT were significantly associated with the presence of residual PH. The stepwise backward multivariate model associated HRR and Jamieson-Type with the hemodynamic outcome 1 year post-PEA. Intriguingly, no such association were found for the Δ HR or HRR during CPET (Table 4).

## Discussion

In the current study we could show for the first time that additional characteristics derived from 6MWT in operable CTEPH were of clinical relevance. The novel findings of the present study include 1) that EID during 6MWT is commonly observed in operable CTEPH pre-PEA, and 2) baseline HRR, but not EID, during 6MWT is associated with the hemodynamic outcome 1 year post-PEA. To the best of our knowledge, this is the first study that evaluated specific characteristics from 6MWT in a selected cohort of operable CTEPH patients and demonstrated their impact on the hemodynamic outcome post-PEA.

CTEPH patients with reduced exercise capacity, mostly in WHO functional class III and severely impaired pulmonary hemodynamics prior PEA were prospectively included. Our cohort demonstrated the typical precapillary PH pattern with significant impairment of RV function resulting in a reduced CI, elevated PVR and mPAP [7, 30–33]. Hemodynamic parameters and exercise capacity substantially improved 1 year post-PEA, albeit 21 patients (56.8 %) presented with a residual PH. In total, a significant improvement of the functional outcome 1 year post-PEA was observed as described previously [3, 4]. However, the high rate of residual PH in our selected cohort underlined that the individual outcome post-PEA differed while rates of residual PH up to 35 % were previously reported [20, 32, 34].

Our results describe for the first time that after exclusion of obstructive pulmonary diseases, almost ~32 % of operable CTEPH exhibited a substantial EID during 6MWT prior to PEA. Further, these patients reported an enhanced, albeit not significant, increased sensation of dyspnea during 6MWT. Our data support the high prevalence of EID during 6MWT in CTEPH as described previously in a heterogeneous group of PH patients [35]. Furthermore, patients with residual PH showed significantly higher BORG scores and lower peak SpO_2_-levels 1 year post-PEA. The cause of EID and sensation of dyspnea is multifactorial in CTEPH, including gas exchange abnormalities with exertional hypoxaemia due to increased V_D_/V_T_ and V/Q mismatch, increased chemosensitivity as a stimulus of exercise hyperventilation and insufficient enhancement of CO due to RV dysfunction [16, 28, 36]. One can speculate that ventilatory inefficiency with an increased V_D_/V_T_ during exercise due to increased V/Q mismatch results in EID, which however couldn’t be extrapolated by our data as significant correlations between EID and V_D_/V_T_ were lacking. The absence of significant associations between EID and V_D_/V_T_ might be related to the relatively small increase in V_D_/V_T_ during exercise observed in our cohort, as previously peak V_D_/V_T_ up to 50 were reported [36]. In addition, baseline resting V_D_/V_T_ was lower than previously reported indicating that pulmonary perfusion was, overall, slightly better in our cohort [30]. Surprisingly, baseline EID was neither associated with hemodynamic parameters 1 year post-PEA nor with the presence of residual PH. Therefore our data emphasized that baseline EID failed as a surrogate marker to predict small-vessel arteriopathy, non-removable material or impaired RV reverse remodeling post-PEA which were identified as major causes of residual PH [20]. Nevertheless, EID was described as a powerful prognostic marker of overall survival in PAH, pulmonary fibrosis or COPD previously [11, 12, 37]. In addition, SpO_2_-levels or EID derived from CPET failed as relevant prognostic factors in our multivariate model.

Interestingly, our data indicated for the first time that ∆HR prior PEA was related to PVR 1 year post-PEA in operable CTEPH patients. In addition, HRR during 6MWT was associated with the presence of residual PH. Even though the association of an enhanced baseline HRR with the presence of residual PH might look paradoxical at first sight, its’ pathophysiologic meaning in CTEPH is explicable. In general, exercising PAH patients exhibit a limited increase in stroke volume due to systolic and diastolic impairment, increased RV afterload and impaired ventriculoarterial coupling [38], while the diastolic pressure–volume relationship determines filling and CO [39]. In CTEPH the chronic obstructions in the pulmonary circulation lead to an increase in RV afterload and eventually to an impairment in RV function [40] such that the increase in CO is compensatory achieved through increases in HR. The disproportionate, albeit enhanced ∆HR at baseline inside a cohort with an overall impaired HRR, may reflect the severity of the disease. One can speculate that an enhanced HRR at baseline was mainly mirroring the inadequate response of the RV to adapt to higher load during exercise with a further impaired overall RV function and advanced RV remodeling. Therefore, HRR was associated with the hemodynamic outcome 1 year post-PEA in patients that were less prone to RV reverse remodeling. PEA immediately reverses the increased RV afterload in CTEPH patients, while the magnitude of reverse RV remodeling after PEA has been shown to correlate with changes in hemodynamics, restoration of ventriculoarterial coupling or reduction of RV systolic wall stress [33, 40–43]. However, it has been proposed that RV remodeling is only partly reversible because of diffuse myocardial fibrosis and differed individually [40]. As previously reported, regression of concentric hypertrophy and restoration of full right and left systolic function assessed by means of Cardiac Magnetic Resonance Imaging (cMRI) require a longer period of time of up to several years [43].

Interestingly, the ∆HR and the HRR during CPET failed as prognostic marker in our cohort, highlighting the value of 6MWT in CTEPH. Being a self-paced submaximal effort test, it results in an aerobic steady-state, as opposed to CPET which is a maximal effort test. Thus differences in HRR with increased HR in more severely ill patients, as discussed above, may be observed in this test, but not at maximal effort. We therefore speculate that ∆HR and the HRR assessed during 6MWT are superior in reflecting disease severity in a selected cohort of operable CTEPH patients. As shown previously, differences of the cardiac, ventilatory and metabolic response during 6MWT in comparison with CPET in patients with PAH are occurring [15, 44]. CPET is associated with higher a minute ventilation, respiratory exchange ratio and maximal HR in comparison with 6MWT [44]. Therefore, Deboeck and coworkers concluded that 6MWT is a more realistic test for the determination of the exercise capacity than CPET and is more reflective of therapeutic interventions [44].

Limitations of the study are the small sample size and highly selected patient sample that excluded patients with obstructive pulmonary diseases. As we are a national referral center, the 1 year follow-up visit was not mandatorily performed in our center and accounts for the dropout rate of 26 patients. Furthermore, 31 patients refused participation at the time of screening due to various reasons. Taking together this drop out rate produces potential bias. In addition, since the rate of residual PH was higher than reported in the literature, a selection bias is possible. To confirm and elucidate the pathophysiological findings of our study, larger prospective studies including post-procedural cMRI and angiographies for quantification of reverse remodeling and residual perfusion impairments will be necessary.

## Conclusions

This is the first prospective study to describe an association of additional characteristics derived from 6MWT with the hemodynamic outcome 1 year post-PEA in a selected cohort of operable CTEPH patients. Our preliminary results indicate that ∆HR and HRR assessed during 6MWT are of clinical value in patients with operable CTEPH, and HRR during 6MWT may serve as a predictor of residual PH. EID was commonly observed in operable CTEPH, albeit failed as a significant prognostic factor.

## Abbreviations

6MWD: six-minute walking distance
6MWT: six-minute walking test
CI: cardiac index
cMRI: cardiac magnetic resonance imaging
COPD: chronic obstructive pulmonary disease
CPET: cardio-pulmonary exercise testing
CTEPH: chronic thromboembolic pulmonary hypertension
EID: exercise-induced oxygen desaturation
FEV1: forced expiratory volume in 1 s
FEV1/VC: forced expiratory volume in 1 s/vital capacity
FRC: functional residual capacity
H-FABP: heart-type fatty acid-binding protein
HR: heart rate
HRR: heart rate reserve
IQR: interquartile range
mPAP: mean pulmonary arterial pressure
NT-proBNP: N-terminal fragment of pro-brain natriuretic peptide
PAH: pulmonary arterial hypertension
PASP: pulmonary artery systolic pressure
PAWP: pulmonary arterial wedge pressure
PEA: pulmonary endarterectomy
PH: pulmonary hypertension
PVR: pulmonary vascular resistance
RAP: right atrial pressure
RHC: right heart catheter
RHC: right heart catheterization
RV: right ventricle
SD: standard deviation
SpO_2_: oxygen pulse saturation
TAPSE: tricuspid annular plane systolic excursion
TLC: total lung capacity
VC: vital capacity
V_D_: absolute dead space
V_D_/V_T_: dead space ventilation
VO_2_: oxygen uptake
WHO: World Health Organization
ΔHR: heart rate response
